# Anti-Stemness and Anti-Proliferative Effects of Cadmium on Bovine Mammary Epithelial Cells

**DOI:** 10.3390/vetsci12010007

**Published:** 2024-12-29

**Authors:** Penggang Liu, Xueli Chen, Yuqing Zhao, Waseem Ali, Tianle Xu, Jing Sun, Zongping Liu

**Affiliations:** 1 College of Veterinary Medicine, Yangzhou University, Yangzhou 225009, China; sherley0418@163.com (X.C.); yuqingzho124@gmail.com (Y.Z.);; 2 Jiangsu Co-Innovation Center for Prevention and Control of Important Animal Infectious Diseases and Zoonoses, Yangzhou University, Yangzhou 225009, China; 3 Joint International Research Laboratory of Agriculture and Agri-Product Safety of Ministry of Education of China, Yangzhou University, Yangzhou 225009, China; tl-xu@yzu.edu.cn

**Keywords:** gli1 signaling pathway, apoptosis, cadmium, regulation

## Abstract

Cadmium accumulation in the body can damage a variety of organs and impair their development and functions. In the present study, we investigated the effect of cadmium on the stemness and proliferation of normal bovine mammary epithelial cells (BMECs). Normal bovine mammary epithelial cells treated with cadmium chloride were assessed for the expression of stemness-related proteins and cell proliferation. Western blotting results found that exposure to different concentrations of cadmium (0, 1.25, 2.5, and 5 μm) for 48 h significantly increased Gli1 expression but unexpectedly decreased the expression of downstream stem cell-related proteins including BMI1, SOX2, and ALDH. However, we also observed that treatment with 5 μm cadmium for 48 h inhibited mammosphere formation using microscopy. In this study, cadmium exposure significantly reduced cell viability and mobility. Flow cytometry detection found that cadmium decreased the percentage of cells in the G0 phase but increased the percentage of cells in the S phase and the apoptosis rate. Furthermore, cadmium exposure significantly increased the levels of caspase-8, caspase-3, and PARP cleavage as assessed by western blotting. Our study uncovers a previously unrecognized role of cadmium in mammary cell stemness and suggests that cadmium may affect breast development by impairing normal stem cell self-renewal and inducing their apoptosis. Therefore, this study provides important scientific significance regarding whether heavy metal cadmium affects normal breast development.

## Simple Summary

Cadmium is a non-essential trace element in the body. Excessive cadmium intake in animals and humans can lead to abnormalities in function and metabolism. Prior studies suggest that heavy metal pollution may cause the abnormal development of mammary glands. This study focuses on the effects of cadmium on the proliferation of bovine mammary epithelial cells (BMECs) and the mechanisms by which it induces apoptosis.

## 1. Introduction

With the development of modern industry and agriculture, the problem of heavy metal pollution has become increasingly prominent and has received increasing attention. Heavy metals enter the animal body through a variety of routes, then bioaccumulate in the animal body, and affect the food chain through biological magnification. Heavy metals are difficult to degrade after entering the body, causing toxicity to all systems and organs of the body. Their half-life is long. Once heavy metals enter the animal body, their harmful effects persist for a long time, and some even cannot be completely discharged from the body. However, cadmium is one of the toxic heavy metals that affects the health of human animals. Cadmium has estrogen-like activity similar to estradiol that activates high expression levels of factors such as estrogen receptor α (ERα) and G protein-coupled receptor (GPR30). These receptors play pivotal roles in regulating gene expression and cellular processes linked to cell proliferation [1,2,3]. Dietary intake of cadmium worldwide ranges from 0.12 to 0.49 μg/kg per day, with higher intakes observed in children aged 1 to 6 years. Survey data indicate that lactating infants consume between 0.37 and 0.49 μg/kg per day, primarily due to the higher concentration of cadmium in mammary milk. These levels can even exceed the World Health Organization’s weekly cadmium intake standards, posing a serious threat to the health of infants and young children [4,5]. Moreover, studies have shown that low cadmium levels in dairy cow milk are positively associated with milk production, as this toxic metal is easily absorbed by the mammary glands and enters the food chain [6]. Research has found a significant decrease in the twin birth rate of cows exposed to high cadmium levels compared to those in low-cadmium environments [7]. The fertilization rate of artificial insemination in these cows is also significantly reduced, weakening their breeding performance. Furthermore, daily cadmium intake from feed and drinking water exceeding 30 mg/kg results in decreased milk production and increased milk fat content [8].

Excessive intake of the metal cadmium in animals can cause abnormal expression of signaling pathways that regulate cell proliferation. Studies have shown that acute or chronic cadmium exposure promotes high expression of stem cell factors such as Shh, Gli1, SOX2, and Nanog in prostate epithelial cells, as well as increases their ability to invade and migrate [9]. Wei et al. [10] reported that long-term exposure to cadmium can stimulate cell proliferation, adhesion, migration, invasion, and tumor cytoskeleton remodeling in triple-negative mammary cancer cells. Furthermore, exposure to large amounts of cadmium during embryonic development can lead to Shh signaling disorders and abnormal development of thymocytes [11]. The study data indicate that excess cadmium activates the Shh signaling pathway, resulting in abnormal cell proliferation and leading to the abnormal development of tissue and organs [12]. Therefore, we aim to explore whether cadmium interferes with the cell cycle of mammary epithelial cells through the Shh signaling pathway and its downstream stem cell-related protein expression. This research may provide insights into mechanisms that may affect the normal development of the mammary gland.

Experiments have shown that cadmium chloride disrupts the cell cycle of cow mammary epithelial cells and significantly promotes apoptosis in these cells. Cadmium treatment in mouse models has resulted in notable mammary damage, characterized by thickening of the glandular follicular wall, apoptosis, and inflammatory exudation and infiltration [13]. Li et al. [14] has proposed that cadmium ions enter cells through Ca^2+^ channels, activating calpain, which induces DNA fragmentation and apoptosis. Additionally, cadmium stimulates caspase-8, directly activating effector caspases-3 and -7, thereby altering the biochemical and morphological characteristics of cells to trigger apoptosis. In human early childhood leukemia cells (HL-60) treated with cadmium, cytochrome c (Cyt c) is released and acts as an effective activator of caspase-9 in the cytoplasm, leading to the subsequent activation of downstream effector caspase-3. Cadmium ions can also accumulate in mitochondria, causing mitochondrial swelling and triggering mitochondria-dependent apoptosis [15,16]. However, limited research has been conducted on the effects of cadmium on mammary epithelial cells in cows.

## 2. Materials and Methods

### 2.1. Antibodies and Reagents

GLI-1(C-1) (SC-51575, 1:1000 dilution), snail (SC-271977, 1:1000 dilution), Nanog (SC-374001, 1:1000 dilution), and actin (SC-517582, 1:1000 dilution) antibodies were purchased from Santa Cruz (Dallas, TX, USA). Bmi1 (6964, 1:1000 dilution), PARP (9532, 1:1000 dilution), caspase-8 (4790, 1:1000 dilution), cleaved caspase-8 (8592, 1:1000 dilution), caspase-3 (14220, 1:1000 dilution), cleaved caspase-3 (9661, 1:1000 dilution), MMP-9 (13667, 1:1000 dilution), OCT-4 (75463, 1:1000 dilution), and SOX2 (3579, 1:1000 dilution) antibodies were purchased from Cell Signaling Technology (CST) (Danvers, MA, USA). Cadmium chloride (CdCl_2_, 202908) was purchased from Sigma Aldrich (St. Louis, MO, USA). Secondary antibodies were obtained from Jackson Immuno-Research (Philadelphia, PA, USA). RPMI-1620 medium, Trizol, streptomycin, L-glutamine, and fetal bovine serum (FBS) were obtained from Thermo Fisher Scientific (Waltham, MA, USA). Cell Counting Kit-8 (CCK-8) and ethylene diamine tetraacetic acid (EDTA) were purchased from Beyotime (Shanghai, China). Radioimmunoprecipitation assay buffer (RIPA buffer) was purchased from (20101ES60, NCM, Suzhou, China).

### 2.2. Cell Culture

The bovine mammary epithelial cell (BMEC) line, derived from cow mammary epithelial cells and subject to long-term preservation in a −80 °C refrigerator, was used in this experiment. BMECs were cultured in RPMI-1620 medium supplemented with 10% fetal bovine serum (FBS), 1% streptomycin, and 1% L-glutamine. The cells were maintained in an incubator at 37 °C with a 5% CO_2_ atmosphere. After a period of cell growth, the cells were washed with PBS, and 400 microliters of pancreatic enzymes were added for digestion. The experimental cells were then cultured as planned.

Cadmium chloride was obtained (CdCl_2_, 233-296-7, Sigma, Darmstadt, Germany). In vitro experiments were designed using different cadmium chloride concentrations (0, 1.25, 2.5, and 5 μM) and different exposure times (0, 16, 24, and 48 h) to treat BMECs. Cells were treated with CdCl_2_ across a time (0, 16, 24, and 48 h) and concentration gradient (0, 1.25, 2.5, and 5 μM). After treatment, the cells were washed with PBS, followed by the addition of 1 mL phosphate buffered saline (PBS) and 10–20 μL EDTA. Then, the cells were incubated at 37 °C for 3–5 min. The cell suspension was collected in EP tubes and centrifuged at 8000× *g* for 5 min at high speed to obtain cell pellets. Subsequently, 2× loading buffer and cell lysis buffer were added to the pellets, followed by sonication for 20 s. The samples were boiled at 95 °C for 5 min to complete cell lysis, and the final cell sample was prepared.

### 2.3. Immunoblotting Analysis

After CdCl_2_ treatment, cells were washed three times with PBS. Then, RIPA lysate buffer was added. The supernatant was collected as the extracted protein sample. This protein extract was mixed with 6× loading buffer, denatured at 95 °C for 7 min, and then separated on a 5% SDS-PAGE gel. Gel electrophoresis was performed using a randomized sampling principle (Millipore Corporation, Billerica, MA, USA).

The proteins were transferred to a PVDF membrane and incubated with primary antibodies: mouse monoclonal anti-cyclinD1 (DCS-11, Invitrogen, 1:800 dilution), rabbit polyclonal antibody Nanog (ab80892, abcam, 1:600 dilution), rabbit poly-clonal antibody NF-κB (64921SF, Cell Signaling Technology (CST), 1:1100 dilution), and mouse monoclonal antibody Bax (ab3191, abcam, 1:1000 dilution) overnight at 4 °C. The membrane was washed with TBST four times (4 min/time) and reacted with HRP-labeled secondary antibody (1:1000) for 1.5 h at room temperature. The protein bands were detected using an ECL kit (NCM Biotech, Shanghai, China) and photodensitometry (Bio-Rad) with β-actin.

### 2.4. Aldefluor Analysis

BMECs were cultured in six-well plates. After CdCl_2_ was added, the cell density increased to 1 × 10^6^. The cells were washed with PBS and incubated with 30 μL of EDTA in a 37 °C incubator for 5 min. The sample was centrifuged in an EP tube. Then, 0.8 mL assay buffer was added to the suspension to maintain cell activity. Then, 5 μL of activated buffer was added, and the sample was subject to a quick vibration on the vortex oscillator. Then, 500 μL of the heavy suspension was added to the brown EP tube (5 μL DEAB) and mixed. The samples were incubated for 45 min in a 37 °C cell incubator and centrifuged. Then, the activated buffer was removed. Cells were then transferred to a cell flow tube for onboard testing.

### 2.5. Cell Viability and Scratch Experiments

Defrost Cell Titer-Glo buffer was equilibrated at room temperature. When the cell density in the 96-well plate reaches 2000~3000, CdCl_2_ at different concentrations is added for 48 h. Then, 100 μL buffer is added, and the plate is covered with aluminum foil. The plate is shaken for 2 min on the shaker, and the data are obtained using an enzyme meter.

The cells in the 12-well plate grow to 70~80% confluence. A yellow pipette tip was used to scratch the bottom of the cell plate, and CdCl_2_ and medium were added after PBS washing. A fixed-time microscope takes pictures before and after cadmium exposure, and the software ImageJ (v1.8.0.345) is used to measure cell gaps and assess the effect of drugs on cell mobility.

### 2.6. Mammosphere Analysis

Under normal conditions, BMEC cells are cultured. When the cell density reaches 80%, the BMECs are digested by digestive enzymes and seeded into a low attachment six-well plate specifically designed to inhibit cell attachment and facilitate the growth of non-adherent mammospheres. Then, BMECs were treated with 5 μM CdCl_2_ for 48 h. After treatment, cells are digested with trypsin, and 5000 cells are seeded into a mammosphere culture plate. Then, 3 mL of B27-supplemented DMEM/F12 medium is added along with 20 ng/mL each of epidermal growth factor (EGF) and fibroblast growth factor (FGF). Images are captured, and the number of mammospheres is counted on day 9 and 15.

To calculate the number of mammospheres under the inverted optical microscope, a marking line is used to measure the diameter of the microsphere. A microsphere has a diameter of 50 μm or greater and includes about 100 cells, and this cell mass counts as 1 microsphere. The number of mammospheres is calculated using the cell count method.

### 2.7. Flow Cytometry Assays

Cells are treated with cadmium. To detect the cell cycle, collect cells using trypsin and fix in 70% ethanol at 20 °C for 12 h. Cells are washed twice with PBS buffer and incubated with PI/RNase staining buffer in the dark for 20 min. Then, the cell cycle was determined using a flow cytometer. Each experiment was repeated at least 3 times.

The cells were cultured in a 12-well plate with CdCl_2_ for 48 h. After processing the cells, 100 μL of 1 × binding buffer was added, and the solution was mixed by vortexing. Then, 2.5 μL fluorescein isothiocyanate (FITC) was added to 15 μL of the experimental sample, and the sample was incubated for 5 min before adding PI. The cells of the control group were treated according to the experimental requirements, and all samples were transferred to the flow tube, and 1 × binding buffer was added. The experimental sample was tested. Each experiment was repeated at least 3 times.

### 2.8. Data Statistics and Analysis

The optical density of the western blot results was measured using Image-Pro Plus 6.0 software. Experimental data were analyzed using SPSS 21.0 software, and results are presented as mean ± SEM (standard error of the mean). In vitro studies were repeated at least three times. Statistical analysis was performed using independent sample t-tests to compare differences between samples, and one-way ANOVA with LSD (least significant difference) was used to analyze differences among multiple groups. Significance levels were defined as follows: *p* < 0.01 denotes a very significant difference, *p* < 0.05 denotes a significant difference, and *p* > 0.05 denotes no significant difference.

## 3. Results

### 3.1. CdCl_2_ Activates the SHH Signaling Pathway and Reduces Stem Cell-Associated Protein Expression

**Figure 1:**
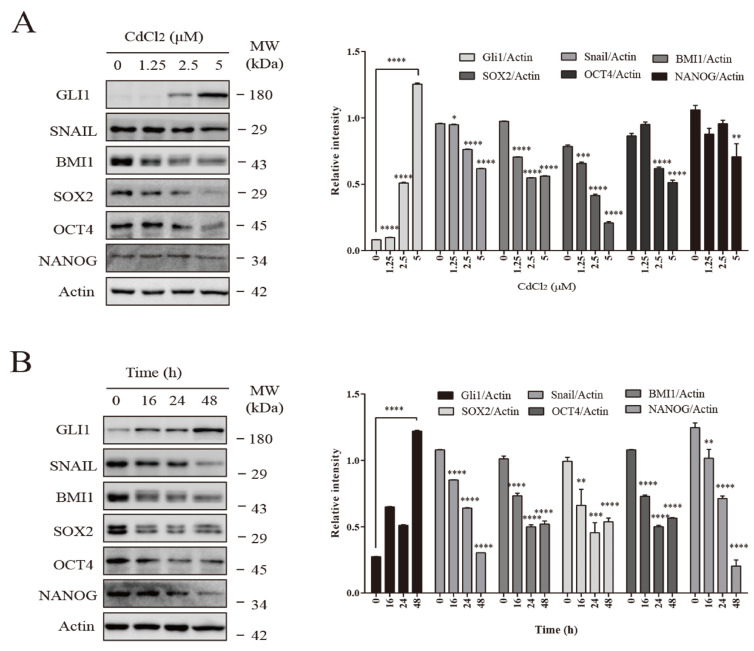
Effects of cadmium on the Shh-Gli1 signaling pathway and stem cell marker proteins in BMECs. (**A**) BMECs were treated with CdCl_2_ at different concentrations (0, 1.25, 2.5, and 5 μM) for 48 h. (**B**) BMECs were also treated with CdCl_2_ (2.5 μM) for different time intervals (0, 16, 24, and 48 h). * *p* < 0.05, ** *p* < 0.01, *** and **** *p* < 0.001. (File S1).

BMECs were treated with CdCl_2_ at different concentrations (0, 1.25, 2.5, and 5 μM) for 48 h, and BMECs were also treated with CdCl_2_ (2.5 μM) for different time intervals (0, 16, 24, and 48 h). As shown in Figure 1A,B, CdCl_2_ increased the protein expression of Gli1 in a concentration- and time-dependent manner, while the levels of stem cell-associated proteins, including SNAIL, BMI1, SOX2, OCT4, and NANOG were reduced.

### 3.2. Cadmium Inhibits Self-Renewal of Mammary Epithelial Stem Cells

**Figure 2:**
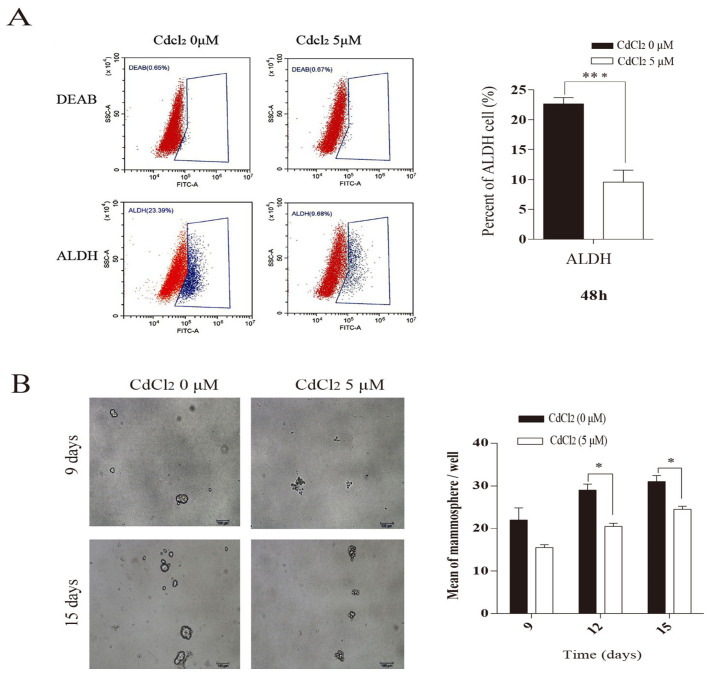
Effects of cadmium on ALDH and the number of microspheres formed by BMECs. (**A**) Effects of cadmium on ALDH in BMECs. (**B**) Effects of cadmium on the number of BMEC microspheres. Red for tumor cells and blue for tumor stem cells. * *p* < 0.05, *** *p* < 0.001.

The present study examined the content of the stem cell marker aldehyde dehydrogenase (ALDH). As shown in Figure 2A, the ALDH-positive cell percentage in the CdCl_2_-treated group was 9.68%, while the ALDH-positive cell percentage in the control group was 23.39%. CdCl_2_ significantly decreased the percentage of ALDH-positive cells in mammary epithelial cells.

In further investigations, BMECs were treated with 5 μM CdCl_2_ for 48 h. Then, the medium was replaced with serum-free medium for 9 and 15 days. The cadmium chloride-treated group exhibited a significant reduction in both the size and number of mammary spheroids compared to the control group (Figure 2B).

### 3.3. Cadmium Reduces Mammary Epithelial Cell Viability and Interferes with Mammary Epithelial Cell Cycle Processes

**Figure 3:**
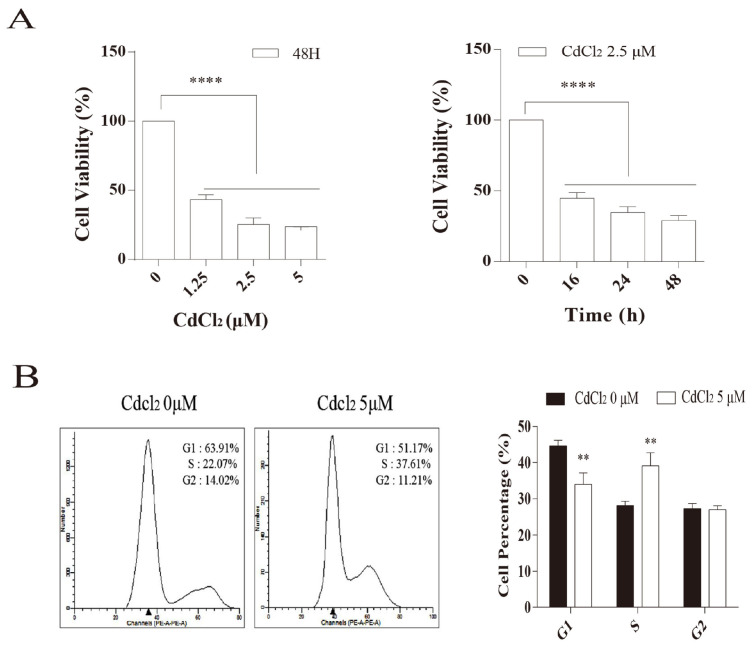
Effects of cadmium on cell viability and the cell cycle of mammary epithelial cells. (**A**) The effect of cadmium on the viability of BMECs. (**B**) The effects of cadmium on the cell cycle of BMECs. ** *p* < 0.01, **** *p* < 0.001.

During the experiment, equal volumes of Cell Titer-Glo™ reagents were added to the cell culture medium, and the luminescent signal was proportional to the ATP levels, which in turn were positively correlated with the number of viable cells. As shown in Figure 3A, there was a concentration- and time-dependent decrease in BMEC viability.

PI is an intercalating nucleic acid fluorescent dye that can specifically bind to cellular DNA, and the fluorescence intensity is positively correlated with PI binding. This allows for the detection of cellular DNA content. BMECs were treated with CdCl_2_ at concentrations of 0 and 5 μM for 24 h to investigate the effects of cadmium on the cell cycle. As shown in Figure 3B, the proportion of cells in the G1 phase was significantly decreased in the CdCl_2_ -treated group (63.91% to 51.17%), while the proportion of cells in the S phase was significantly increased (22.07% to 37.61%), resulting in a prolongation of the cell cycle. These findings indicated that CdCl_2_ interferes with the normal cell cycle progression of mammary epithelial cells.

### 3.4. Cadmium Reduces the Migration of Mammary Epithelial Cells

**Figure 4:**
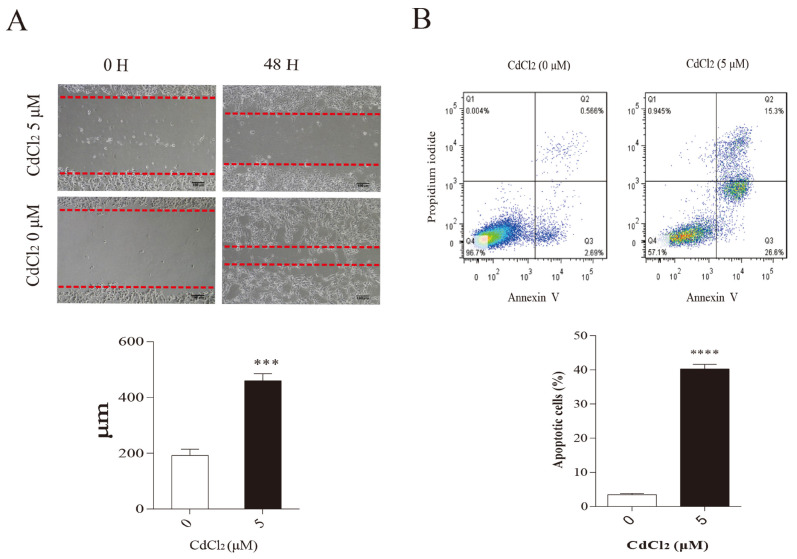
Effects of cadmium on mammary epithelial cell migration. (**A**) Effects of cadmium on the migration of BMECs. The distance between the red dotted lines is the migration distance of the cells. (**B**) Effects of cadmium on BMEC apoptosis. *** and **** *p* < 0.001.

After exposing BMECs to 5 μM CdCl_2_ for 48 h, micrographic images were taken to observe changes in cell gaps. As illustrated in Figure 4A, after the control group of cells grew for 48 h, the scratch generated in the cell layer almost disappeared. The distance of the scratch in the cell layer in the CdCl_2_ treatment group was significantly wider, and the distance between the red dotted lines was measured. A small number of dead cells was observed. This indicates that CdCl_2_ impairs the migration of mammary epithelial cells.

A flow cytometry assay for apoptosis was conducted after exposing BMECs to 5 μM CdCl_2_ for 48 h. The results demonstrated that the cadmium-treated group had a higher Annexin V positivity rate compared to the control group (Figure 4B). This suggests that cadmium induces apoptosis in BMECs.

### 3.5. Cadmium Induces Apoptosis in Mammary Epithelial Cells

**Figure 5:**
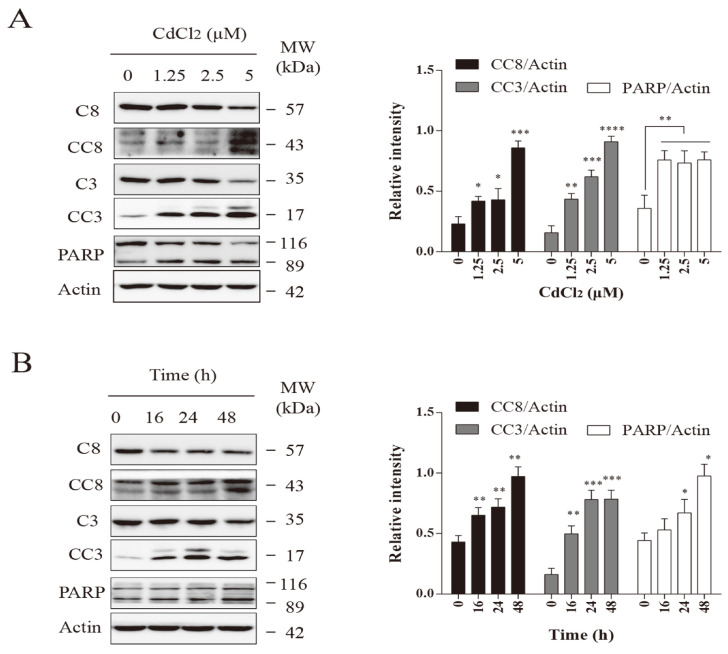
Effects of cadmium on BMEC apoptosis. (**A**) BMECs were treated with various concentrations of CdCl_2_ (0, 1.25, 2.5, and 5 μM) for 48 h. (**B**) BMEC cells were also treated with 2.5 μM CdCl_2_ for different time periods (0, 16, 24, and 48 h). * *p* < 0.05, ** *p* < 0.01, *** and **** *p* < 0.001. (File S1).

In Figure 5, BMECs were treated with various concentrations of CdCl_2_ (0, 1.25, 2.5, and 5 μM) for 48 h, and BMEC cells were also treated with 2.5 μM CdCl_2_ for different time periods (0, 16, 24, and 48 h). The results indicate that CdCl_2_ promotes the activation of caspase-8 and caspase-3 as well as PARP cleavage, suggesting an induction of apoptosis.

## 4. Discussion

Bovine mammary epithelial cells (BMECs) contain stem cells with self-renewing and multi-directional differentiation properties. These mammary epithelial cells promote remodeling of the bovine mammary epithelium and are essential for studying the physiological mechanism of the mammary gland [17]. The Shh signaling pathway has been found to play a role in regulating the self-renewal of normal stem cells such as those found in the hematopoietic system, skin, nervous system, and mammary glands [18]. The Shh-Gli1 signaling pathway induces stem cell-associated proteins. BMI1, SOX2, NANOG, and OCT4 expression regulate mammary stem cell self-renewal [19]. Evidence has shown that the effects of Shh signaling on mammary stem cell self-renewal may be mediated by BMI1. In addition, the Shh-Gli1 pathway induces the expression of the transcription factor SNAIL not only to regulate the epithelial mesenchymal transition (EMT) process, but also to promote the self-renewal of tumor stem cells [20].

Our experimental findings showed that BMECs treated with cadmium chloride exhibit an activated Shh signaling pathway. Under the action of high concentrations of cadmium, it promoted the transformation of normal mammary epithelial cells to tumor cells. However, other downstream stem cell marker proteins, such as BMI1 and SOX2, did not increase. We suspect that because cadmium is a cytotoxic substance, treatment with a high concentration leads to the death of a large number of cells. Stem cells are a small part of the mammary epithelial cells. When a large number of cells die, the stem cell content decreases [21,22,23]. High concentrations of cadmium were found to significantly reduce ALDH levels, consistent with previous results. In order to further determine whether cadmium kills cells by promoting apoptosis or necrosis, the programmed necrosis marker mixed lineage kinase domain-like protein (MLKL) was first tested, and the protein phosphorylation level was not significantly increased [24,25,26]. However, the cleavage levels of the apoptosis proteins, caspase-8, caspase-3, and PARP, were found to be significantly higher. These findings suggest that cadmium causes apoptosis, which in turn leads to reduce stem cell content. This is consistent with the high levels of cadmium in the literature that can induce apoptosis [27,28].

This toxic effect of cadmium on the mammary epithelium can seriously affect the development of the mammary gland. Experiments have shown that cadmium chloride can induce apoptosis and interfere with the cell cycle of cow mammary epithelial cells [29]. In addition, the mammary glands of cadmium-treated mice were significantly damaged, and the glandular follicular wall was significantly thickened. Moreover, glandular follicular cell apoptosis as well as inflammatory exudation and infiltration were observed [13]. This experiment also confirmed the cytotoxic effects due to cadmium. High concentrations of cadmium cause mass apoptosis and a decrease in cell viability. In addition, cell migration is weakened, the cell cycle is disturbed, and the number of mammospheres is significantly reduced.

Mouse experiments have demonstrated that the heavy metal cadmium exhibits estrogen-like activity in the body, leading to an increase in the number of solid areas and terminal buds in the mammary during puberty, as well as a reduction in the number of glandular follicular shoots [30]. For instance, the population of cells forming the mammary bud expands, resulting in an increase in both the number and density of mammary branch sites and epithelial cells. These changes further contribute to the expansion of the mammary stem cell population. The increase in basal cells and ER α may potentially lead to the malignant transformation of the mammary glands [31,32]. Cadmium exposure can also result in morphological changes in mammary glands in mammals. These changes include the remodeling of mammary tissue, which is characterized by increased fat content, reduced activity of mammary gland epithelial cells, and changes in the emulsion concentration. Additionally, cadmium exposure simultaneously reduces calcium levels in the β-casein gene and mammary tissue [33]. Consequently, cadmium disrupts mammary function, impairing the development of mammary glands, which are important in the feeding of offspring.

Rat experiments have confirmed that cadmium induces apoptosis in primary cortical neurons by activating caspase-8, Jun N-terminal kinases (JNK), and mitochondrial apoptosis pathways. The knockout of Fas enhances cellular activity in the presence of cadmium and inhibits apoptosis by blocking cadmium-induced activation of Fas, caspase-8, and JNK [34]. When mice were orally administered cadmium chloride at a dosage of 1.5 mg/kg for 35 days, high expression of apoptosis-related genes and proteins, such as caspase-1, caspase-3, caspase-8, and caspase-7, was activated, leading to restricted growth and impaired myocardial microstructure in the mice [35]. Another study found that the accumulation of cadmium in the bodies of freshwater mussels increases over time and is capable of activating caspase-3, caspase-8, caspase-9, and Ca-ATPase; this accumulation also caused ultrastructural changes and DNA fragmentation [36]. Current research revealed that cadmium chloride exhibited a concentration gradient and time gradient, increasing the phosphorylation of caspase-8 and caspase-3 and the cleavage of PARP, thereby promoting apoptosis. According to reports, early exposure of zebrafish to cadmium severely affects their normal growth and development and increases oxidative damage and cellular apoptosis. Additionally, cadmium activates cysteine aspartate-specific proteases (caspase-3) and induces apoptosis by altering the expression of key genes, including pro-apoptotic factors such as Bcl-2-associated X (BAX) and caspase-9, as well as components of death receptor pathways and tumor necrosis factor receptor 1 (TNF-α/TNFR1) and caspase-8 [37,38].

## 5. Conclusions

These experiments demonstrated that high levels of heavy metal cadmium activate the Shh-Gli1 signaling pathway, resulting in increased GLI1 expression. However, this activation is accompanied by reduced expression of stem cell-related proteins and also leads to decreased cell viability, mobility, and tumorigenesis. Additionally, cadmium exposure significantly increased the levels of cleaved caspase-8, caspase-3 and PARP, as confirmed by ALDH testing, which indicated a notable increase in the apoptosis rate. Therefore, these findings suggest that cadmium can inhibit the proliferation of mammary epithelial cells and promote the occurrence of apoptosis.

## Data Availability

The datasets used and/or analyzed during the current study are available from the corresponding author on reasonable request.

## Supplementary Materials

The following supporting information can be downloaded at: https://www.mdpi.com/article/10.3390/vetsci12010007/s1, File S1: Original Western Blot Figures.
