# Supplementary material for: Anti-Stemness and Anti-Proliferative Effects of Cadmium on Bovine Mammary Epithelial Cells
Source: Vet Sci. 2024 Dec 29;12(1):7. doi: 10.3390/vetsci12010007 (PMC11769218; doi:10.3390/vetsci12010007)

3.1. Cadmium activates SHH signaling pathway and reduces stem cell-associated protein expression

1.GLI1-48h

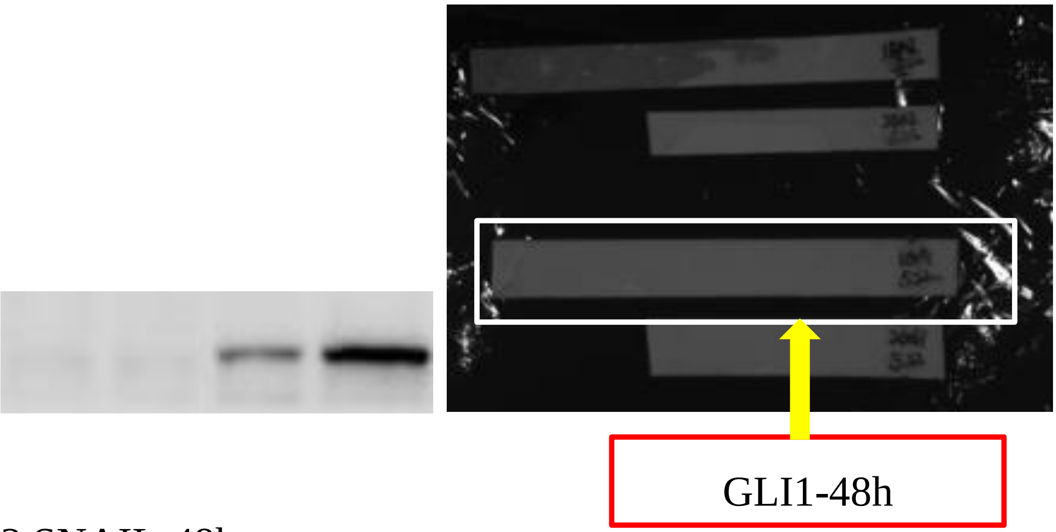

2.SNAIL-48h

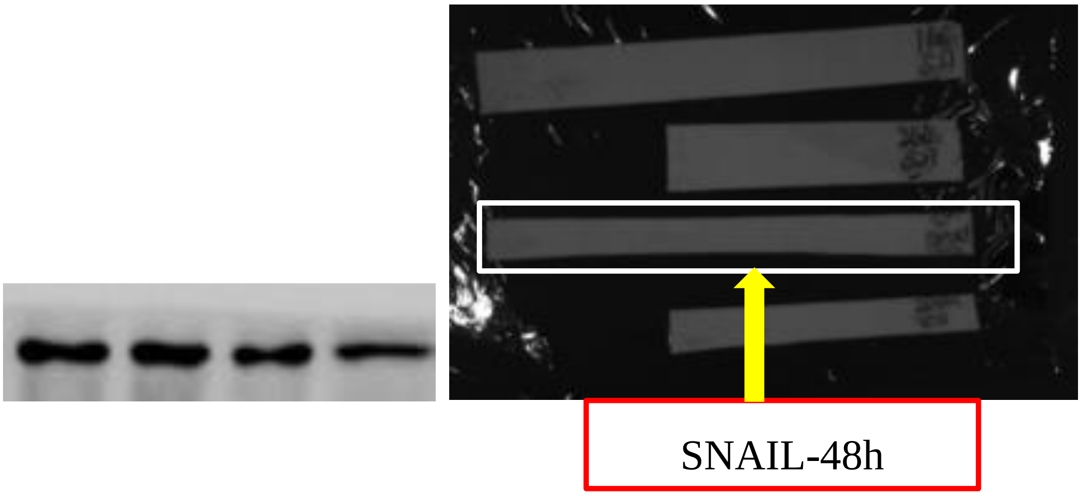

3.BMI1-48h

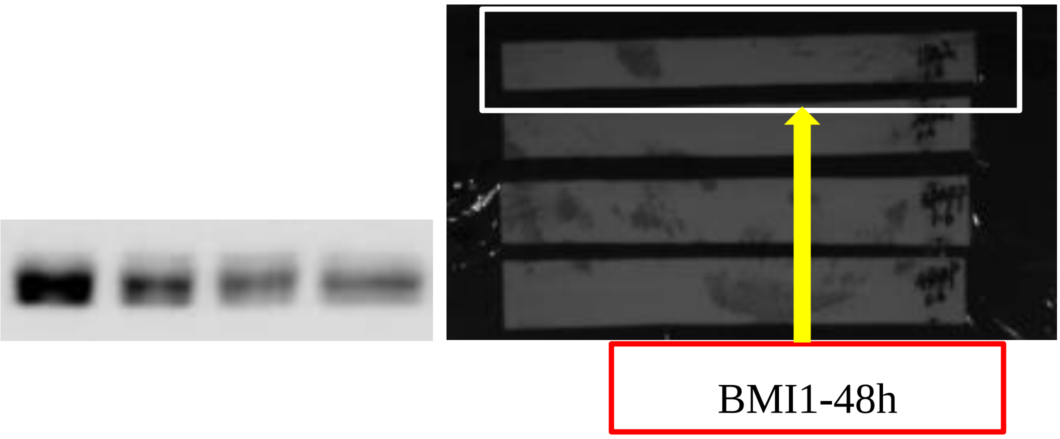

4.SOX2-48h

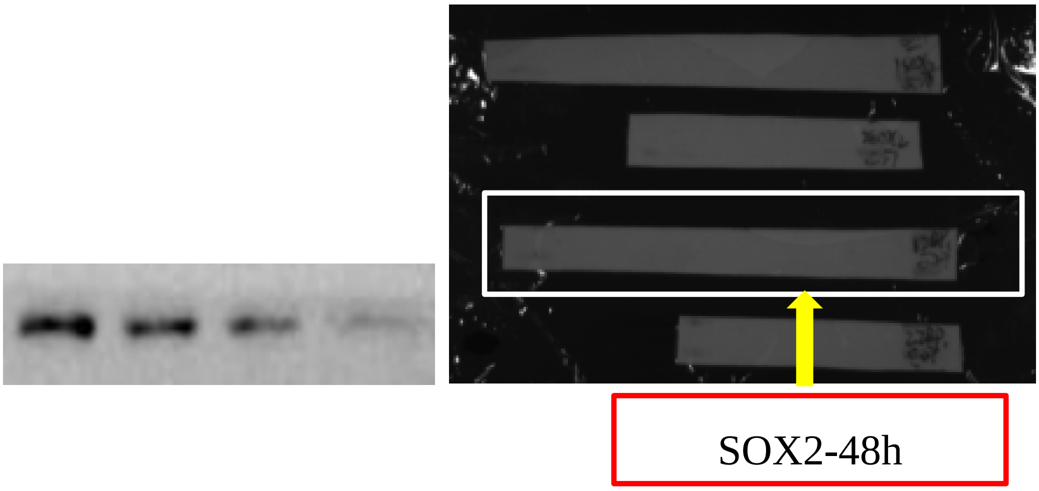

5.OCT4-48h

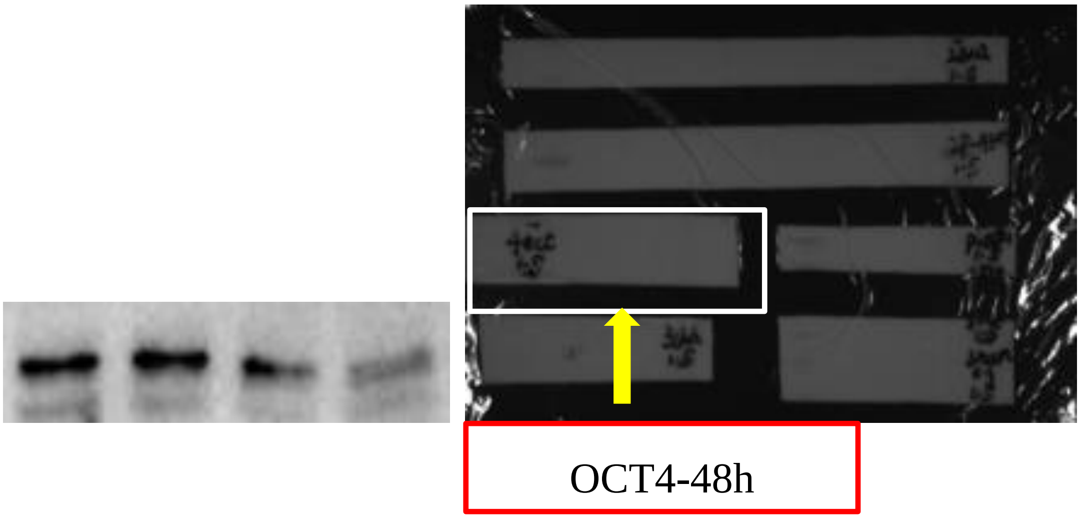

6.NANOG-48h

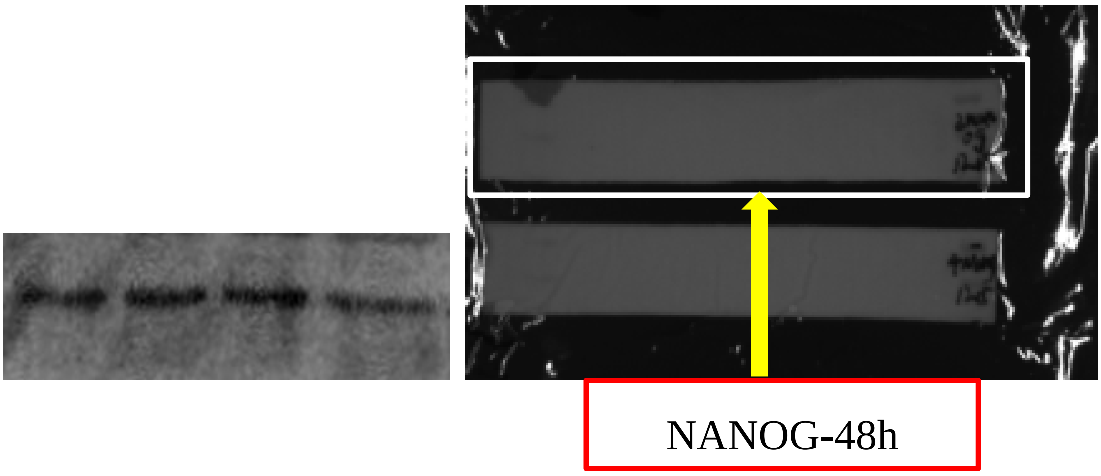

7.Actin-48h

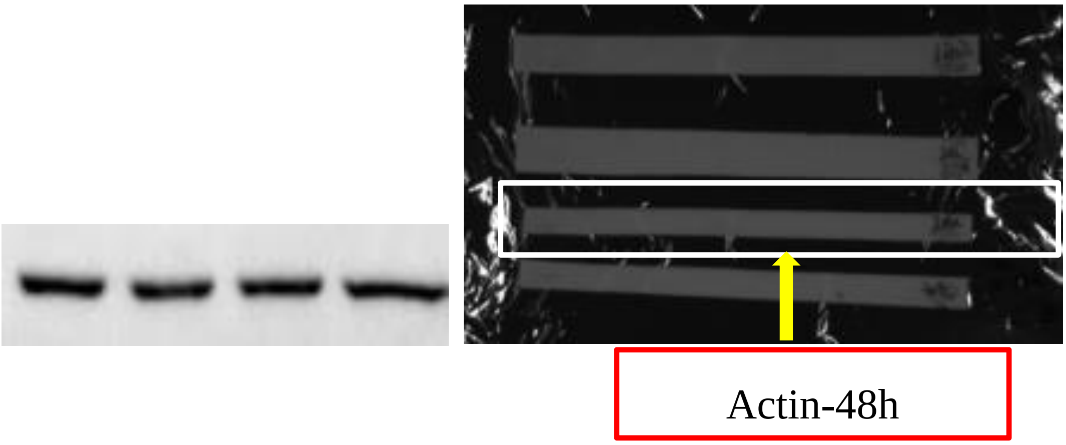

8.GLI1-2.5μM

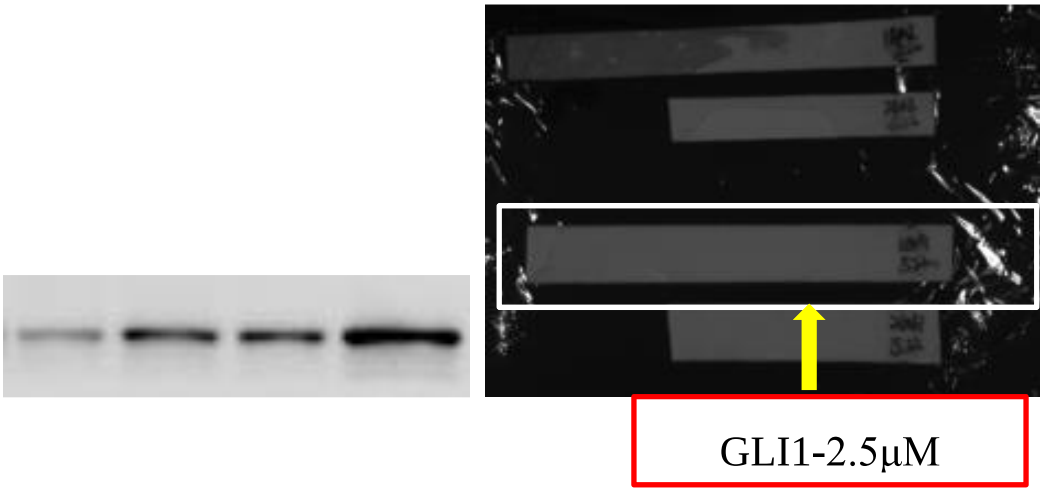

9.SNAIL-2.5μM

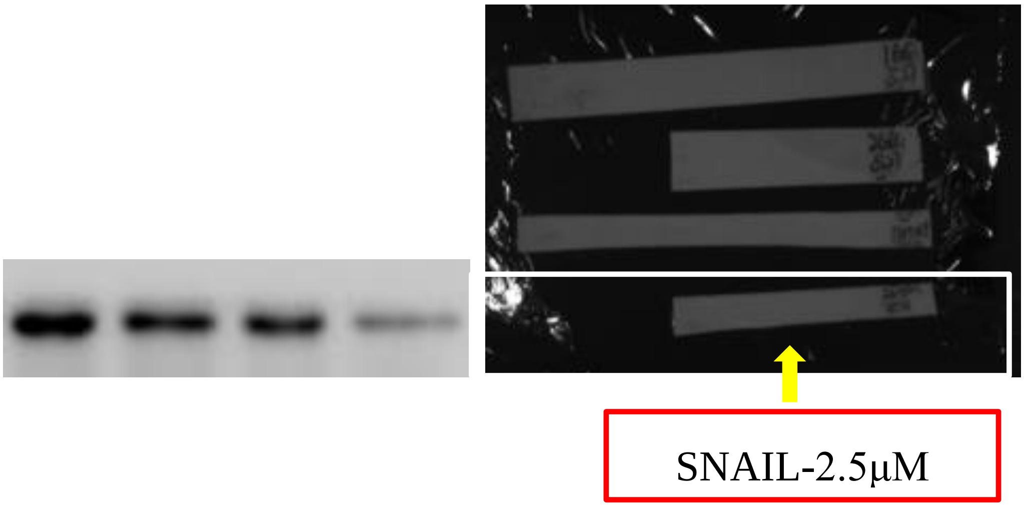

10.BMI1-2.5μM

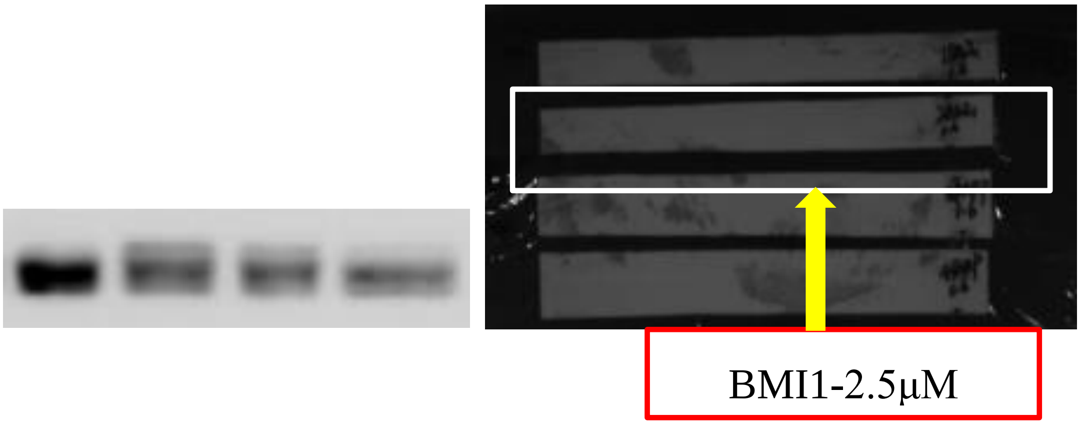

11.SOX2-2.5μM

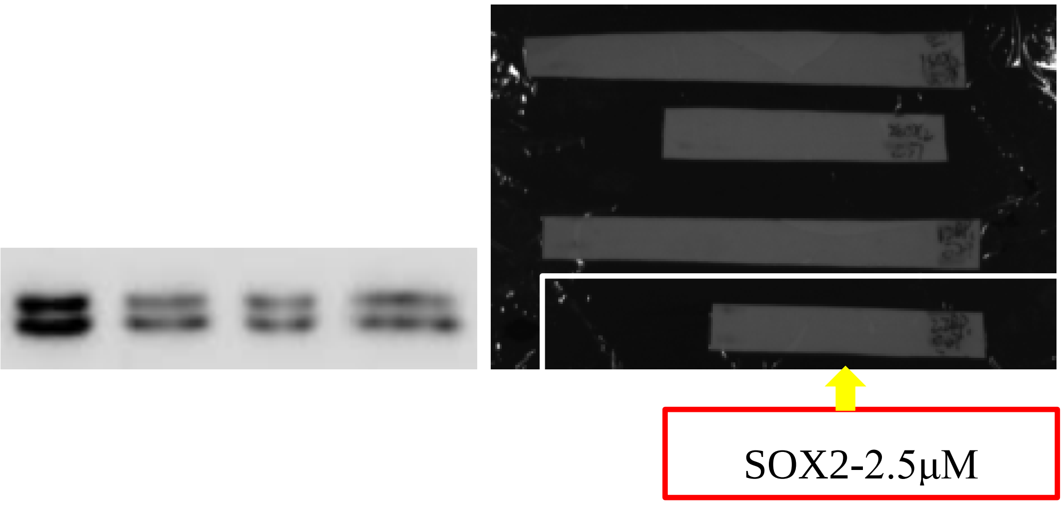

12.OCT4-2.5μM

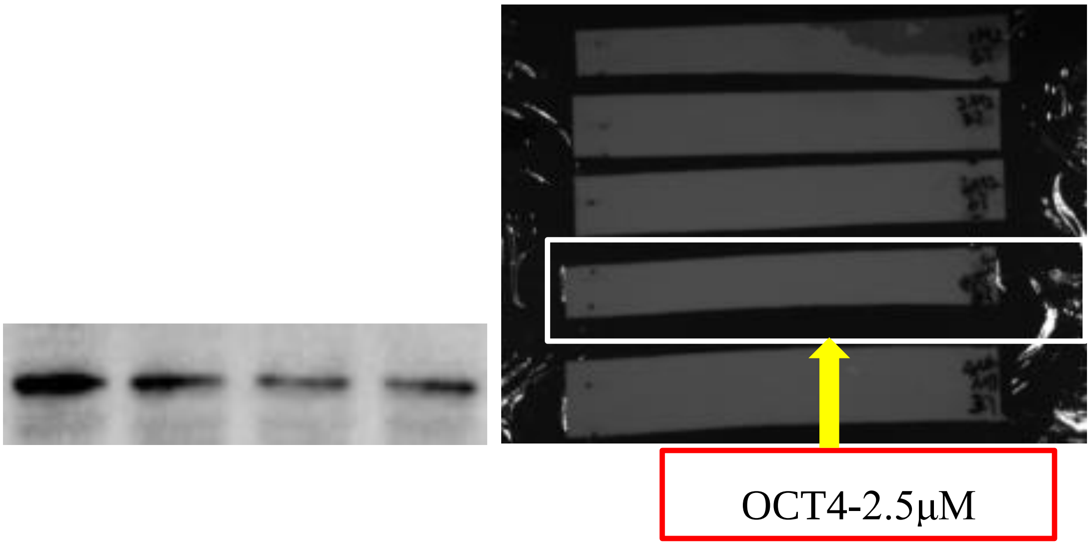

13.NANOG-2.5μM

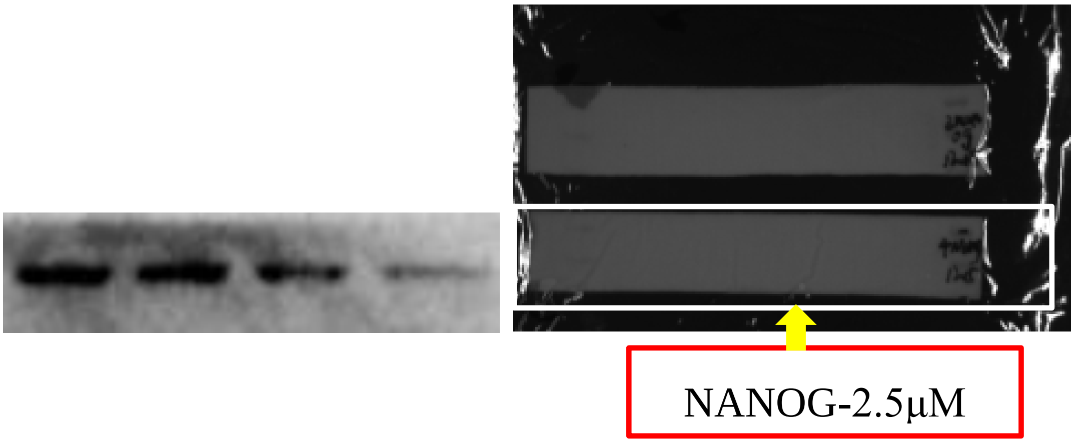

14.Actin-2.5μM

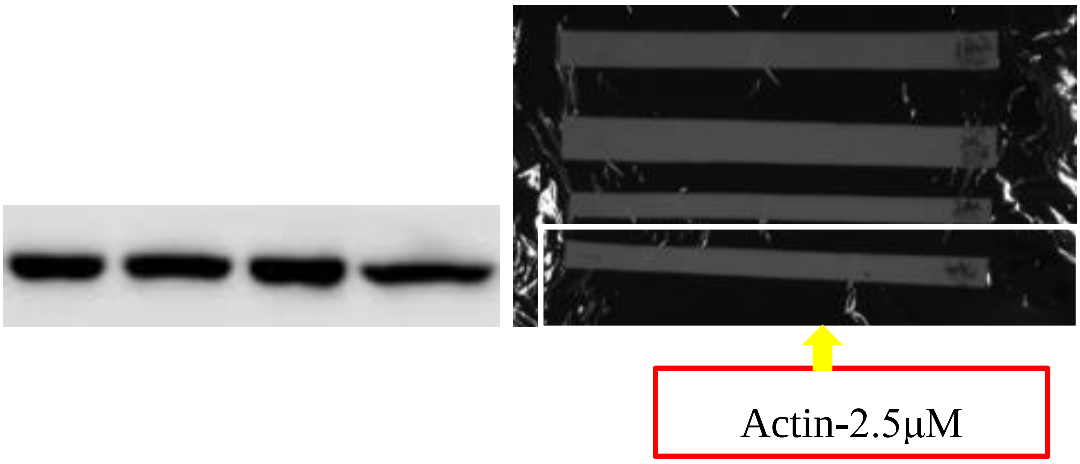

3.5 Cadmium induces apoptosis of breast epithelial cells

1.Caspase 8-48h

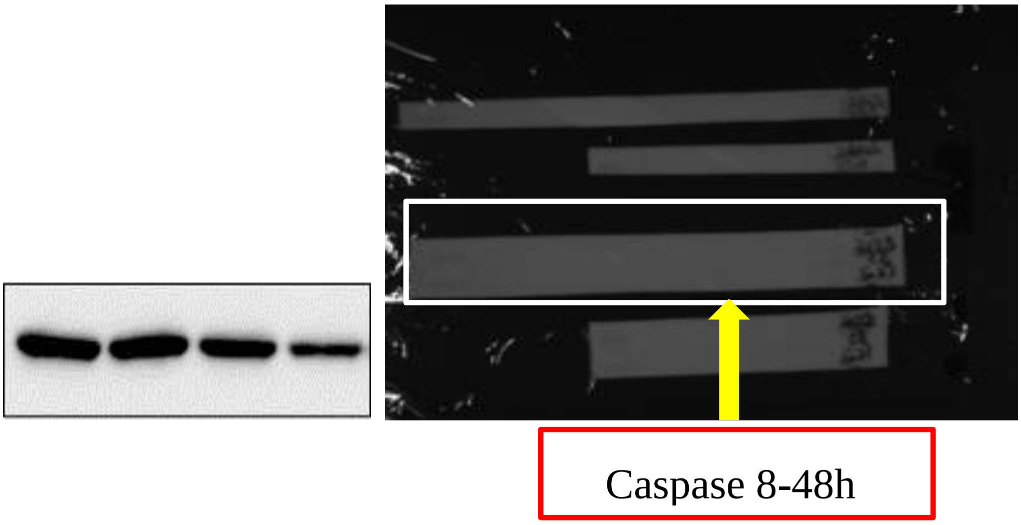

Caspase c8-48h

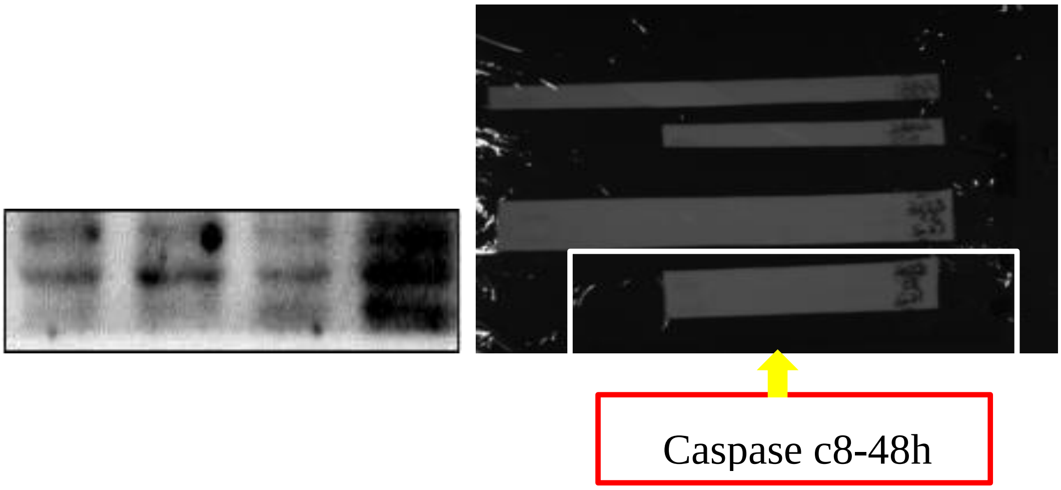

2.Caspase 3-48h

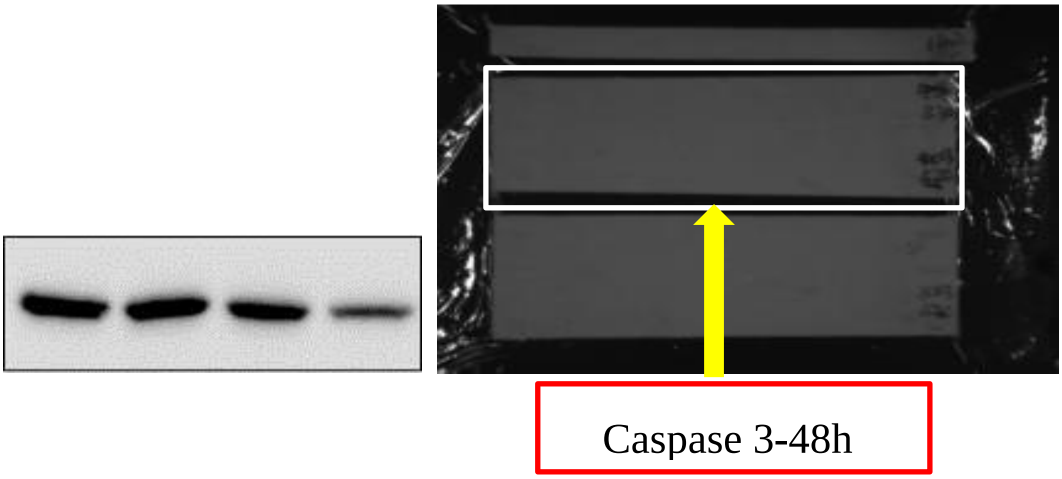

Caspase c3-48h

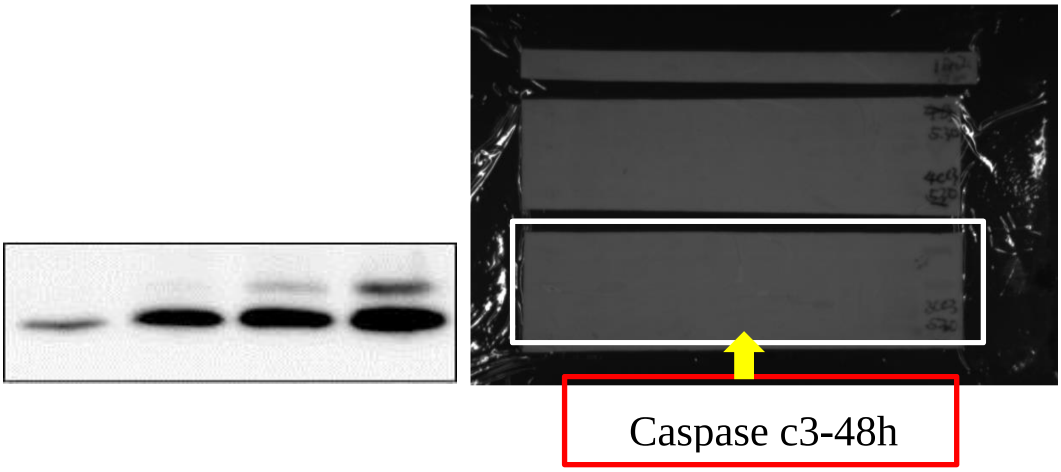

3. PARP-48h

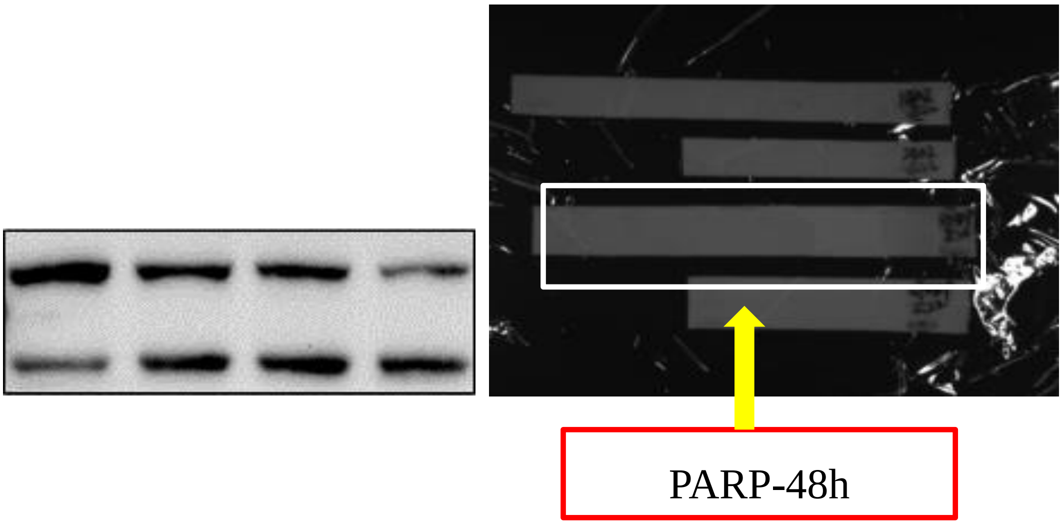

4. Actin-48h

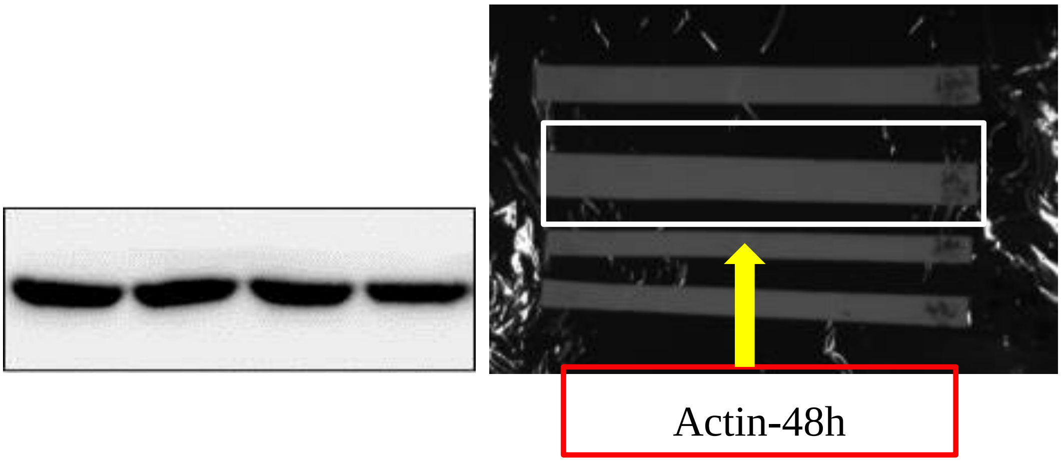

5. Caspase 8-2.5μM

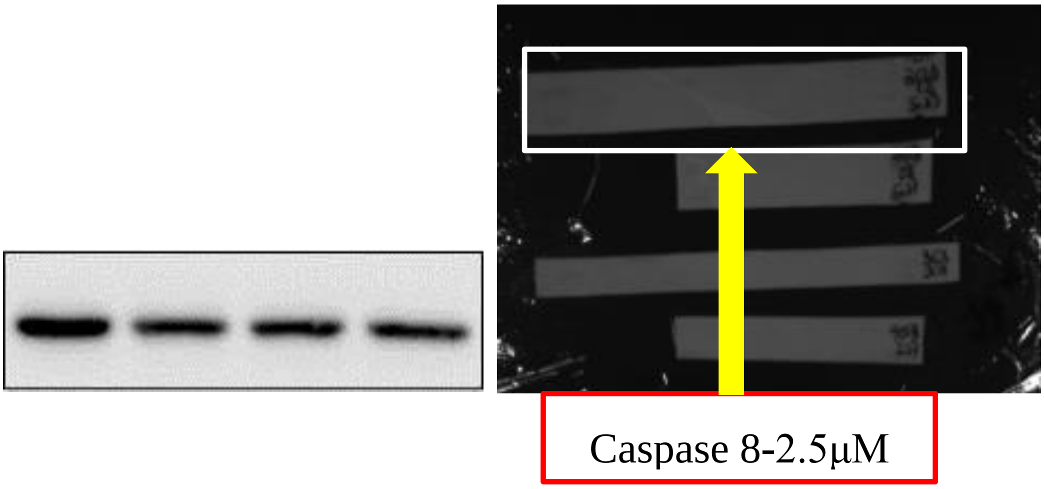

Caspase c8-2.5μM

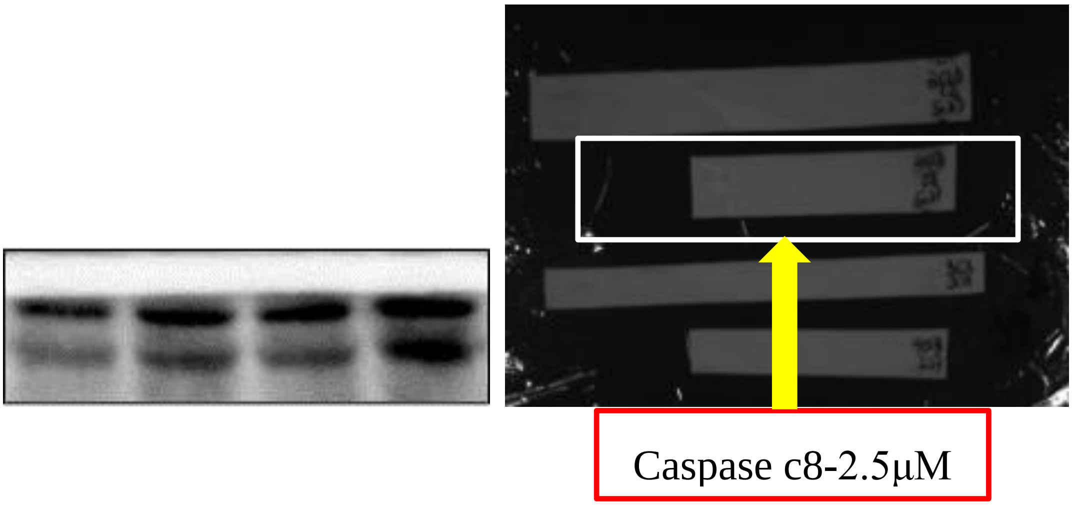

6. Caspase 3-2.5μM

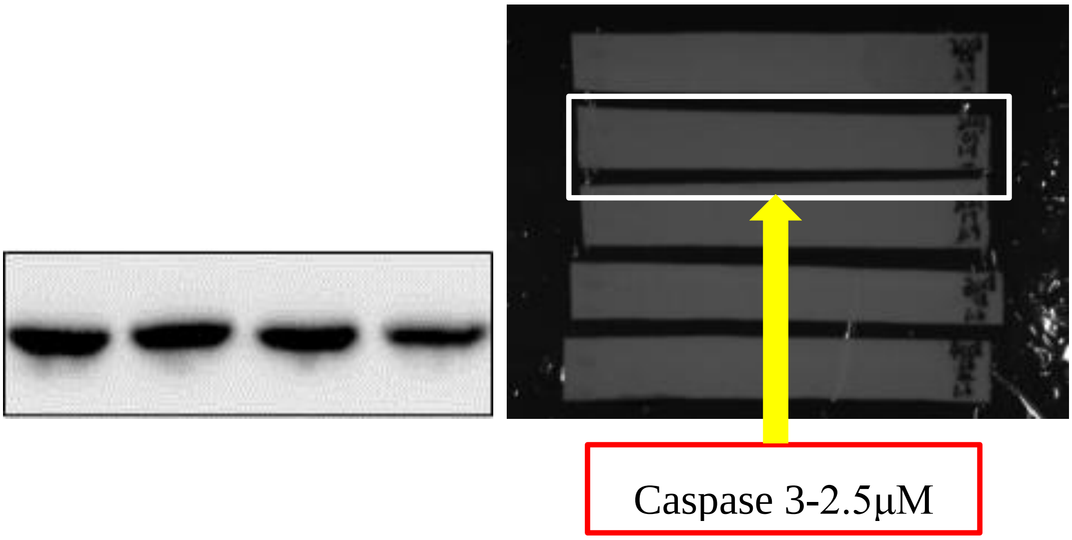

Caspase c3-2.5μM

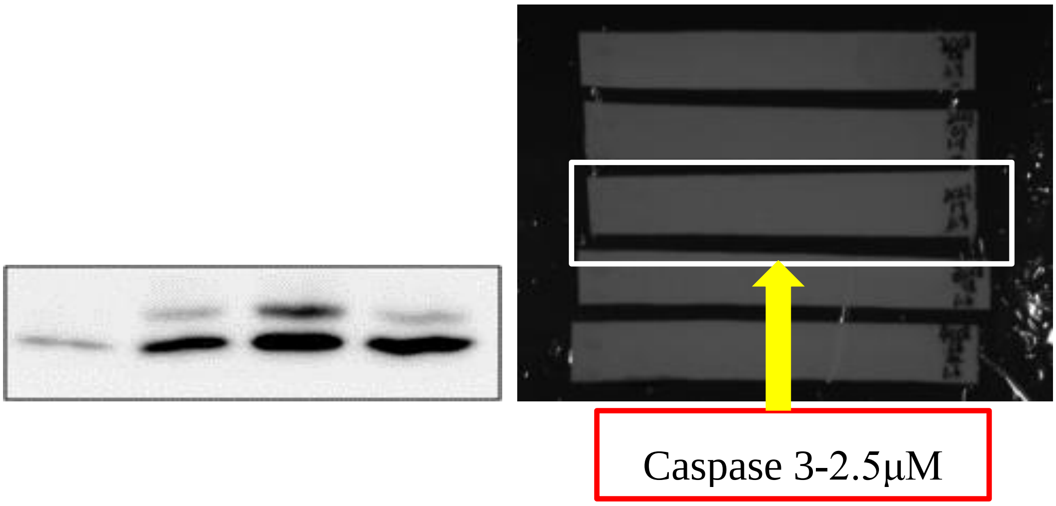

7. PARP-2.5μM

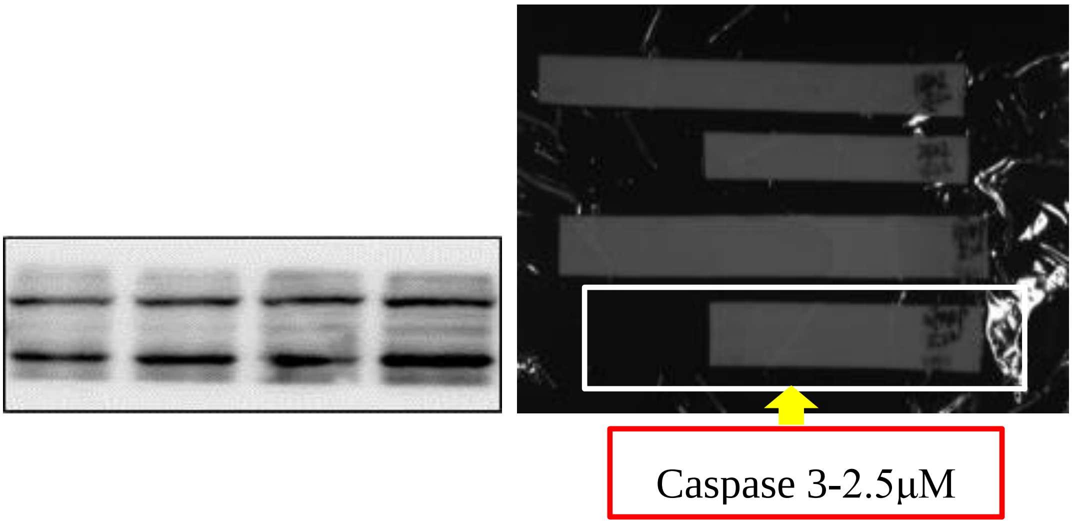

8. Actin-2.5μM

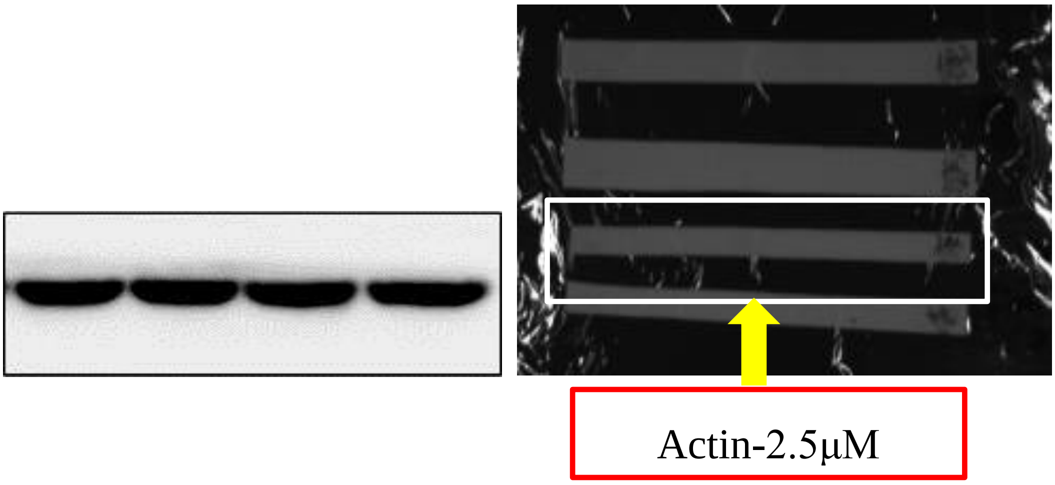

Supplement: Supplementary file 1 [file vetsci-12-00007-s001.zip › vetsci-3369349-supplementary.pdf]
